# Are Jews Smarter Than Everyone Else?

**DOI:** 10.4103/0973-1229.34526

**Published:** 2008

**Authors:** Sander L. Gilman

**Affiliations:** * *Emory University, Atlanta GA 30345, USA*

**Keywords:** Intelligence, Jews, Race, Smartiness

## Abstract

The debate about “race” and “intelligence” seems to be never ending. The “special nature” of the intelligence ascribed to “Jews” has recently reappeared in an essay by one of the authors of the notorious study of race and intelligence – The Bell Curve. How this debate is constructed and what its implications are for the reappearance of “race” as a category in medical and biological science is at the core of this present essay.

## Introduction

In *Commentary* in April 2007, Charles Murray, the coauthor of *The Bell Curve* (1994), again made the claim that “Jews are smarter” than everyone else (Murray, 2007). In *The Bell Curve*, he and his late (and Jewish) coauthor, Richard Herrnstein, first presented their argument about the intellectual superiority of “Ashkenazic Jews of European origins,” who “test higher than any other ethnic group (Murray and Herrnstein, 1994). Jews in America and Britain have an overall IQ mean somewhere between a half and a full standard deviation above the mean, with the source of the difference concentrated in the verbal component.” Murray again makes the argument of an unmistakable “Jewish Genius,” in *Commentary*, but now with an explanation:

Insofar as I am suggesting that the Jews may have had some degree of unusual verbal skills going back to the time of Moses, I am naked before the evolutionary psychologists′ ultimate challenge. Why should one particular tribe at the time of Moses, living in the same environment as other nomadic and agricultural peoples of the Middle East, have already evolved elevated intelligence when the others did not? At this point, I take sanctuary in my remaining hypothesis, uniquely parsimonious and happily irrefutable. The Jews are God's chosen people. (Murray, 2007)

Who could resist such a claim, especially if it clearly echoes the view that Herrnstein and Murray made earlier that “high intelligence also provides some protection against lapsing into criminality for people who otherwise are at risk”? (Murray and Herrnstein, 1994: p235). Jews are smarter and morally better than everyone else. At least they have “smartiness,” a quality analogous to, and proven by, TV comedian Stephen Colbert's parodic “truthiness”: “truth that comes from the gut, not books” (www.m-w.com/info/06words.htm). It isn't really that Jews are smarter than everyone else; it is just what “everyone” believes.

## “Superior” Intelligence, Selective “In-Breeding” and “Jewish” Genetic Diseases

This view of Jewish superior intelligence echoes another recent “philo-Semitic” argument explaining it. In 2006, Gregory Cochran, Jason Hardy and Henry Harpending – all anthropologists at the University of Utah – made quite a splash with a published study which suggested that Jewish “superior” intelligence was the result of selective “in-breeding” (Cochran *et al.,* 2006). Their paper “Natural History of Ashkenazi Intelligence” argued that Jewish intelligence is simply a compensatory genetic error linked to other “Jewish” genetic diseases, like Tay-Sachs, Gaucher's Disease or Fanconi's anemia. As the authors write:

…perhaps most of the characteristic Ashkenazi genetic diseases fall into this category. Selection has imposed a heavy human cost: not crippling at the population level, cheaper than the malaria-defense mutations like sickle cell and G6PD deficiency, but tragic nonetheless. (Cochran *et al.,* 2006)

This sounds like an updated version of the claim that the Parisian neurologist Jean Martin Charcot made to Sigmund Freud in the fall of 1888 about the predisposition of Jews for specific forms of illness, such as diabetes, where “the exploration is easy” you just have to attribute it to intramarriage, to too many cousins marrying cousins (Gelfand, 1988: p574). The Jews may be smart, but the cost is that they are an “ill people.” This is uncomfortable: smarter means more diseased. Smartiness is a sort of compensatory booby prize for a wide range of diseases ascribed to the Jews. Every brilliant Jew is simultaneously a frail Jew, living the life of the mind rather than that of the body (like the stereotypical Eastern European *Luftmensch*, the Jew who lives on nothing and suffers for it).

Now who but me (clearly a “Jew with smartiness”) is going to argue with the claim that Jews are smarter than everyone else, a racial myth that has its origin in the 19^th^ century? Isn't it obvious? Just count the number of. Nobel prizes, violin virtuosi and so forth. Isn't “Jewish” inherited smartiness proven by the accomplishment of individual Jews? How many Jewish Nobel prize winners or their families actually had any of the eight diseases now called “Jewish genetic diseases”? If I had to trade off my Ph.D. against the BRCA 2 gene for breast cancer in my children, would it be worth it? Well, certainly if, as Charles Murray has it, it were part of the Divine Plan.

Yet speaking about the Jews as a biological entity or to use the new/old term “race” is tempting, especially if part of a divinely ordained plan. And yet there is a real disadvantage to this. Francis Collins, head of the American Human Genome Project, recently commented in *The Economist*:

The downside of using race, whether in research or in the practice of medicine, is that we are reifying it as if it has more biological significance than it deserves. Race is an imperfect surrogate for the causative information we seek. To the extent that we continue to use it, we are suggesting to the rest of the world that it is very reliable and that racial categories have more biological meaning than they do. We may even appear to suggest something that I know is not true: that there are bright lines between populations and that races are biologically distinct. (Anon, 2006; p80)

### We Need to Know What We Are and What We Are Not

The desire to draw these “bright lines” is perhaps intrinsic to human nature. The need to define and control, to identify from where succor or fear comes, is built into all social groups as central to their self-definition. We need to know what we are and what we are not.

Now I could loose a screed against the creation of a unitary racial (or ethnic or even genetic) definition that lumps radically diverse people of radically diverse experience and background into a category labeled the “Jews” or even “Ashkenazi Jews.” I could join the late Steven J. Gould (Gould, 1981) among many others to inquire about the relationship between “race” and yet another suspicious category “intelligence” (smartiness writ scientific), but is it worthwhile doing this? When you have two categories, both of which are created by social consensus, defining one in terms of the other leads to pure nonsense, as Murray and Herrnstein (1994) showed in *The Bell Curve*, where the claim was that we could determine who would steal our hubcaps by IQ exams. Sadly, they did not ask whether such tests or claims about the relationship between IQ and race would predict who would loot Enron (Ken Lay – Christian) or Drexel-Burnham (Michael Millken – Jewish). Claims about a Divine Jewish “smartiness” seem just as superficial. Certainly there are corners of the world, such as Australia, where Jews bemoan the absence of “smart Jews.” One can point to the simple fact the 1954 Nobel prize winner Max Born's granddaughter is Olivia Newton-John (her family moved from England to Australia when the actress was five) as evidence that there is a decay of smartiness when one goes South of the equator, at least if you detest the film *Grease* as much as I do. But just who is the German (born in Breslau, then in Prussia, now in Poland) and Jewish (by parentage if not religion) and Scots (he taught for decades at Edinburgh) Max Born? In Born's official Nobel biography, written at the time of his award, he was a “German” physicist who “as [with] so many other German scientists,… was forced to emigrate in 1933….” How odd? And why were German scientists fleeing Germany in 1933? An example of German smartiness?

The argument about Jewish smartiness rests on individual accomplishments, such as Nobel prizes and violin virtuosi of those who are labeled or self-identified as “Jews,” and these individual accomplishments may well be a reflex of a culture of learning and performance by individuals placed in a specific Diaspora situation. As Albert Einstein pointed out a long time ago:

If my theory of relativity is proven successful, Germany will claim me as a German and France will declare that I am a citizen of the world. Should my theory prove untrue, France will say that I am a German and Germany will declare that I am a Jew. (Address, Sorbonne, Paris; Concise Dictionary of Quotations, 1985; p124-125)

Being “Jewish” is only one identity for him, if an important one. He never made the same claim about his rather amateurish but passionate violin playing eliciting the envy of nations. Such categories as “Jewish Nobel prize winners” are used to chronicle accomplishment of individuals, especially at moments when the social situation permits these individuals to excel and a group needs to identify with these accomplishments to answer charges of inherent difference and assumed inferiority.

### Pressure to Excel and Need to Have Heroes

Such smartiness “declines” as the pressure to excel in any identifiable group diminishes and the group ceases to need “heroes.” If this were not the case, the recent rapid decrease in the number of “Jews” who are applying to medical schools (an index of “intelligence” ever since Francis Galton in the 1980s) could mean that Jews may have lost God's grace but perhaps are also becoming less “diseased” or more at risk for criminality as their smartiness declines. Actually what has happened in the United States is that self-identified Jews are fitting more closely into the profile of the American middle class! They are making choices about their professions based on their middle-class identity rather than the desires of their parents. (And the “New Jews,” the Asian-Americans, an equally invented category, are filling the medical schools because of the social pressures that define this as a major route of success in the American Diaspora.) Are the Jews also becoming “healthier”? Ok, maybe I would trade my Ph.D. (“almost a doctor,” as my beloved mother used to say) for a healthier body. It would be simpler than working out.

But there is a larger, certainly less comic aspect to the “bright line” that the claim of Jewish smartiness makes and the role that non-Jews have had in this argument about Jewish superior intelligence ever since the late 19^th^ century. There seems to be value to the claim that Jews are smarter than non-Jews if you are *not* Jewish. Self-identified non-Jews, such as Murray, in the 21^st^ century, continue to make the claim of Jewish smartiness for reasons that have very little to do with philo-Semitism. The categories by which Jews are defined are the special relationship between God and the chosen people (Murray, 2007); or group inheritance genetics (Cochran *et al.,* 2006); or a mix of both as in the so-called “evolutionary biologist” Kevin MacDonald's *A People That Shall Dwell Alone: Judaism as a Group Evolutionary Strategy* (MacDonald, 1994), which argued that Jewish smartiness created and perpetuated anti-Semitism as a means for group, genetic cohesion.

We can think about these functions of Jewish smartiness in terms of what the psychoanalysts call “projective identification.” I so admire (or fear) someone that I wish to be exactly like them. Yet I know that it is impossible for me to become them (as they are genetic or divinely “different”); so there is always a gap between my “real” self and my desire. And that gap between who I am and what I wish to be means that I must generalize about universals that capture all difference (philo-Semitism). Thus the gap between who I am and who I want to become can never be bridged, as I can never become “Jewish” in this absolute sense. All Jews are… If being Jewish means joining a peoplehood and a religious practice, no such barriers exist. They only exist in the absolute fantasy of inherent Jewish difference, even if this difference is defined in terms of “smartiness.”

## Evangelical Christianity and Judaism

Something very similar takes place within Evangelical Christianity, which envies the Jews but detests Judaism – envies the Jews because they were God's first “chosen people” and are necessary for the plan of the *End of Days*; but detests Jewish religious practice because the Jews continue to deny Christ and will convert at the end of days or be damned. No matter what, like Murray, the Evangelicals can never “be” Jews; they remain always a poor simulacrum of Jewish belief or, in Murray's case, Jewish “smartiness.” Philo-Semitism creates the Jews as a universal and therefore poisoned category. *All* Jews are… The same happened, by extension, to the Israelis after the Six-Day War: they were all powerful and resistant and ethical; since then and as international sympathy for the Palestinians has grown, when any given Israeli acts against “type,” all are damned as too powerful, too rigid, too corrupt. The stereotype of “Super Jew” has transformed itself into Jewish “barbarity.”

But there is yet another turn of the wheel in the debates about Jewish smartiness. By extension, no individual Jew today can be quite smart enough to fulfill the category of the stereotypical “Smart Jew.” By this argument, if you are not a smart Jew, then you are not much of a Jew at all. The Jews are merely clever or facile or glib or superficial but not really smart! For, as the philo-Semites like Murray and his ilk imply, my insight “understands” the essence of the Jews and thus I am in control of the “bright line” that defines the healthy from the sick, the smart from the merely clever. In the end, says Charles Murray, “I am the smartiest one of all.”

## Concluding Remarks

The reappearance of “race” within the medical and biological sciences attempts to “explain” characteristics ascribed to constructed populations through the new lens of genetics and related sciences. That these older categories have little or nothing to do with the new science is not evident. They carry with them much of the baggage of racism, including the so-called “positive” stereotypes of a group.

### Take Home Message

Don't confuse racial categories with scientific ones. Don't assume that making “positive” comments about a group is necessarily a good thing.

## Questions That This Paper Raises

1. Why do stereotypes such as “smartiness” perpetuate themselves?
2. Can claims about “superior intelligence” be harmful?
3. What does “race” have to do with genetics?
4. What does “intelligence” have to do with race?

## About the Author

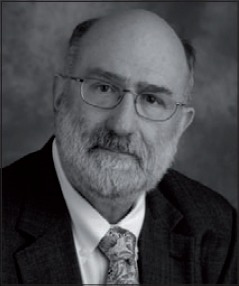



Sander L. Gilman Ph.D., is a distinguished professor of the Liberal Arts and Sciences at Emory University, where he is the director of the Programme in Psychoanalysis, as well as of Emory University's Health Sciences Humanities Initiative. A cultural and literary historian, he is the author or editor of over 70 books. His Oxford lectures “Multiculturalism and the Jews” appeared in 2006; his most recent edited volume, “Race and Contemporary Medicine: Biological Facts and Fictions,” appeared in 2007. He is the author of the basic study of the visual stereotyping of the mentally ill, “Seeing the Insane,” published by John Wiley and Sons in 1982 (reprinted: 1996), as well as the standard study of “Jewish Self-Hatred,” the title of his Johns Hopkins University Press monograph of 1986. He is also on the Honorary International Editorial Board of MSM.
